# Bidirectional association between atopic dermatitis and attention deficit hyperactivity disorder: a systematic review and meta-analysis

**DOI:** 10.1080/07853890.2025.2483370

**Published:** 2025-03-30

**Authors:** Hong-Fei Wang, Shan Liu, Yi Cao, Qiu-Shuang Li

**Affiliations:** a First School of Clinical Medicine, Zhejiang Chinese Medicine University, Hangzhou, China; b Center of Clinical Evaluation and Analysis, The First Affiliated Hospital of Zhejiang Chinese Medical University (Zhejiang Provincial Hospital of Chinese Medicine), Hangzhou, China; c The First Affiliated Hospital of Zhejiang Chinese Medical University (Zhejiang Provincial Hospital of Chinese Medicine), Hangzhou, China

**Keywords:** Atopic dermatitis, attention deficit/hyperactivity disorder, systematic review, meta-analysis, neuropsychiatric comorbidity

## Abstract

**Background:**

Our objective is to elucidate the reciprocal association between atopic dermatitis (AD) and attention deficit hyperactivity disorder (ADHD) by prespecified subgroups and determine potential modified factors.

**Materials and Methods:**

Adhering to PRISMA 2020, we conducted a comprehensive database search up until March 11, 2024. Observational studies reporting on AD and ADHD as either exposure or outcome variables were included. A random-effects model meta-analysis was conducted to calculate pooled estimates. Subgroup and meta-regression analyses were undertaken to explore heterogeneity. Publication bias was investigated *via* funnel plots and Egger’s test.

**Results:**

Overall, 49 studies were determined to meet the inclusion criteria after rigorous screening. Patients with AD were more likely to have ADHD (ORs = 1.34, 95% CI 1.25–1.44, *p* < 0.01; HRs = 1.42, 95% CI 1.20–1.68, *p* < 0.01), while patients with ADHD also had an increased risk of developing AD (ORs = 1.45, 95% CI 1.21–1.73, *p* < 0.01). Subgroup analyses indicated that the associations were particularly pronounced among studies that assessed patients with severe AD (ORs = 2.62, 95% CI 1.76–3.92, *p* < 0.01), suffered from multiple allergic conditions (ORs = 2.89, 95% CI 1.18–7.10, *p* < 0.01) and sleep disturbances (ORs = 2.43, 95% CI 2.14–2.76, *p* < 0.01) simultaneously.

**Conclusion:**

This review substantiates the significant bidirectional association between AD and ADHD, indicating that they serve as mutually independent risk factors and may either exacerbate each other. These findings underscore the necessity for heightened awareness and early targeted interventions, especially in individuals with severe AD manifestations, sleep problems, and multiple allergic diseases.

## 1. Introduction

Atopic dermatitis (AD), a pervasive chronic inflammatory skin disease, afflicts roughly 20% of children and 10% of adults [1]. According to the latest data from the Global Burden of Disease 2019, AD continues to cause significant global morbidity from 1990 to 2019 [2]. Due to its intense itching and recurrent eczematous lesions, it has contributed to an adverse increase in neuropsychiatric issues including depression, attention deficit hyperactivity disorder (ADHD), and suicidal ideation [3,4]. Among them, ADHD ranks as the predominant neurodevelopmental disorder among children [5], with a global prevalence exceeding 5% [6]. In contrast to childhood ADHD, the prevalence of symptomatic adult ADHD is 6.76%, representing a substantial public health burden worldwide [7]. Emerging evidence suggests a bidirectional association between AD and ADHD, underpinned by shared genetic markers and underlying pathophysiological mechanisms (systemic inflammation and immune dysregulation) [8]. While psychostimulants such as methylphenidate have proven effective in managing ADHD, potential side effects like drug misuse and insomnia are concerns [9,10]. Notably, sleep disturbances are a prevalent complaint in both AD and ADHD [11]. This interaction creates a vicious cycle, imposing significant socioeconomic burdens on patients and their families.

Since the 1980s, the association between allergic diseases (including AD, allergic rhinitis, and asthma) and ADHD, whether involving comorbidity or causality, has earned considerable attention from both the public and clinical communities [12,13]. Previous studies have suggested AD could increase ADHD incidence [14–17], while ADHD may influence the onset and progression of AD [18–21]. Pooled analyses also have indicated a positive correlation between atopic diseases and ADHD [22,23], though conflicting results persist [24–26]. Besides, most have concentrated on pediatric populations, neglecting adult populations where AD often persists [23]. Moreover, no comprehensive reviews specifically address the bidirectional relationship between AD and ADHD.

Given the escalating public health burden of AD and ADHD, systematically clarifying this bidirectional relationship is crucial for improving patient screening, treatment, and preventive strategies. Therefore, in this meta-analysis, our objectives are threefold: (1) to elucidate the bidirectional association between AD and ADHD in both pediatric and adult populations; (2) to assess the influence of potential moderating factors (whether age, AD severity and other comorbidities would affect the results); and (3) to ascertain the prevalence regardless of AD as either exposure or outcome variables.

## 2. Methods

Our study followed the Preferred Reporting Items for Systematic Reviews and Meta-Analyses guidelines (PRISMA 2020)[27] and Meta-analysis of Observational Studies in Epidemiology (MOOSE) guidelines [28]. The protocol was pre-registered in PROSPERO (CRD42024521737).

### 2.1. Search strategy

Two reviewers (QL and YC) independently conducted searches of PubMed, Embase, Cochrane Library, Web of Science, PsycINFO, and LILACS from their inception until March 11, 2024. The search terms included ‘atopic dermatitis’, ‘attention deficit hyperactivity disorder’, and related variants. Additionally, we manually scrutinized the reference lists of the included studies and relevant reviews. Details are outlined in Supplement 1.

### 2.2. Study selection

The selected studies were assessed according to the following criteria: (1) peer-reviewed, English-language observational studies (encompassing both retrospective and prospective cohort studies, as well as cross-sectional and case-control studies); (2) studies reporting on AD and ADHD as either exposure or outcome variables (to explore a bidirectional relationship by evaluating the relationship of ADHD in patients with AD compared to those without AD, and vice versa); (3) studies with no age, race, country or AD severity restrictions; (4) AD and ADHD should both be determined with confirmed diagnoses, including clinical diagnosis (clinical criteria, diagnostic codes, medical records), standardized questionnaires, or self-/parent-/caregiver-reported physician diagnosis.

Exclusion criteria were as follows: (1) studies with no valid data could be extracted to compute effect sizes; (2) AD patients with other psychiatric comorbidities like depression, anxiety, and suicide; (3) reviews, letters, commentaries, and conference/meeting abstracts.

### 2.3. Data extraction

Data extraction was carried out by two reviewers (HW and SL), covering details such as first author, publication year, study period, study design, country, sample size, demographics of patients with AD (mean age and gender distribution), method assessment for AD and ADHD, prevalence, and adjusted risk estimates comprising odds ratio (OR), risk ratio (RR), hazard ratio (HR), and corresponding 95% confidence intervals (CIs). When an article provided multiple results, only those estimates with comparable characteristics, specifically the set of confounders adjusted, were extracted. If only information on separate types of ADHD was available, these types were aggregated into a single general ADHD outcome measure.

### 2.4. Quality assessment

The same reviewers utilized the Newcastle-Ottawa Scale (NOS) to appraise each cohort or case-control study [29]. For cross-sectional studies, the Agency for Healthcare Research Quality (AHRQ) criteria were applied [30]. Studies were stratified into low, moderate, or high-quality categories according to their NOS scores (ranging from 0–3, 4–6, to 7–9) or AHRQ scores (ranging from 0–3, 4–7, to 8–11), respectively.

### 2.5. Statistical analysis

Statistical analyses were independently conducted by two reviewers (YC and SL) using Stata version 18.0 (StataCorp, LLC, College Station, TX, authorized by The First Affiliated Hospital of Zhejiang Chinese Medical University). In discussing the mutual association, ORs and HRs from at least two studies were synthesized. ORs and RRs were integrated using the formula: RR = OR ÷ (1 − *p* + (*p* × OR)) [31], where *p* denotes the prevalence of AD in the control group. For prevalence estimates, the denominator was the total number of individuals meeting the AD diagnostic criteria, and the numerator was those diagnosed with ADHD after AD onset when AD was the exposure variable. Conversely, when ADHD was considered as the exposure variable, the denominator was the total number of ADHD and the numerator was those diagnosed with AD after ADHD onset.

Heterogeneity was deemed significant at *I*^2 ^ exceeded 50% or when *P* was below 0.1 in the Q test [32], prompting the application of a random effects model [33]. We conducted sensitivity analyses by sequentially omitting studies, subgroup analyses, and random-effects meta-regressions to examine factors influencing AD and ADHD associations. Subgroup analysis were stratified by geographical region, study type, sample size, study period, diagnostic methods for AD and ADHD, age, gender, AD severity, comorbid conditions. Publication bias was evaluated qualitatively with funnel plots and quantitatively through Egger’s linear regression when at least ten studies were available for meta-analysis. Significant publication bias was identified if the funnel plot was asymmetric or *p* < 0.05 [34]. When data synthesis was deemed inappropriate due to notable clinical or methodological diversity, or a lack of sufficient studies, findings were reported descriptively.

## 3. Results

### 3.1. Characteristics of the included studies

**Figure 1.**
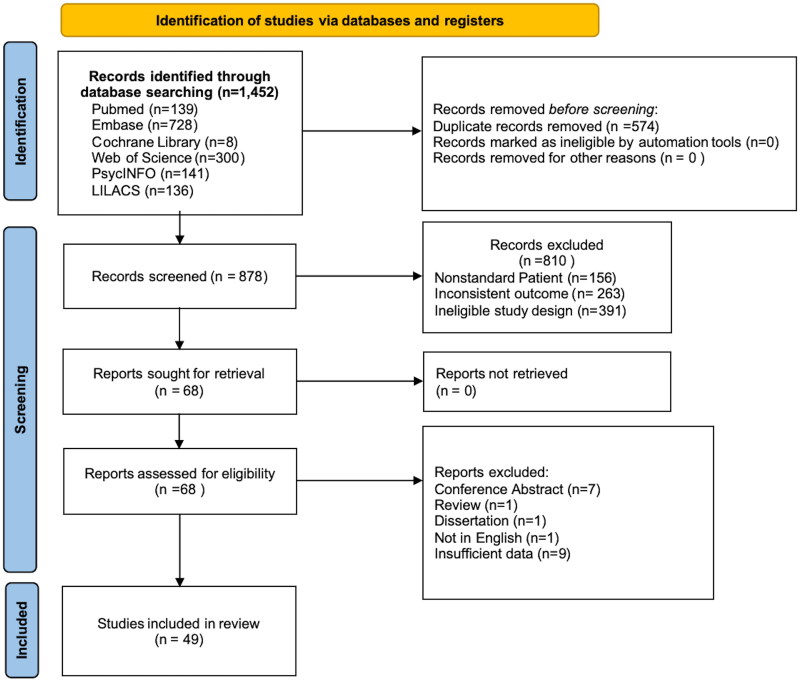
PRISMA flow chart.

From Embase (*n* = 728), PubMed (*n* = 139), Cochrane Library (*n* = 8), Web of Science (*n* = 300), PsycINFO (*n* = 141), LILACS (*n* = 136), a total of 1,452 records were found. After the removal of duplicates (*n* = 574) and studies that did not meet the criteria (*n* = 829), 49 studies were ultimately included. Among these 49 studies, 36 examined ADHD as the outcome with AD as the exposure, while 13 focused on AD as the outcome with ADHD as the exposure (Figure 1).

The key characteristics of 49 studies, comprising 18 cohort studies, 10 case-control studies, and 21 cross-sectional studies, are summarized in Table 1. Between 2009 and 2024, these studies were carried out in Europe (*n* = 17), Asia (*n* = 20), and North America (*n* = 12).

**Table 1.** Characteristics of participants and studies.

(a) ADHD as the outcome with AD as the exposure											
Study	Study type	Country	Study period	No. of participants (AD/non-AD)	Prevalence/Incidence (%)	AD patients’ characteristics		Method assessment		Reported effect	Study quality
						Age, years	Gender (female %)	Method assessment AD	Method assessment ADHD		
Ahn et al. 2019	Cross-sectional	Korea	2002–2014	42641/139486	0.56	NA	NA	ICD 10 code L20.9	ICD 10 F90.0	OR: 1.48 (1.27–1.72)	7
Augustin et al. 2015	Cross-sectional	Germany	2009	30354/262827	8.11	NA	NA	ICD 10 code L20	ICD 10 codes	OR: 1.36 (1.30–1.42)	5
Ballardini et al. 2019	Cohort	Russia	1996–2005	577/11091	NA	NA	NA	ISAAC questionnaire	SDQ	OR: 0.91 (0.77–1.08)	8
Boemanns et al. 2023	Cross-sectional	Germany	2003.5–2006.5	1164/5466	8.10	14.5 ± 2.0	53.8	Parental report of physician diagnosis	Parental report of physician diagnosis	OR: 1.692 (1.253–2.285)	5
Catal et al. 2016	Case-control	Turkey	2013.7–2014.1	80/74	23.80	4.03 ± 1.31	52.5	Hanifin and Rajka classification	ECI-4	OR: 2.57 (1.049–6.298)	4
Genuneit et al. 2014	Cohort	Germany	2000.11–2001.11	200/570	7.50	NA	NA	Parental questionnaires of physician diagnosis and/ or physicians’ reports	Parental questionnaires of physician diagnosis and medication	RR: 5.17 (2.18–12.28)	9
Horev et al. 2017	Case-control	Israel	NA	840/900	7.10	9.48 ± 3.65	49.3	Hospital-based medical records	Hospital-based medical records	OR: 1.79 (1.18–2.73)	7
Hou et al. 2021	Cross-sectional	USA	1997–2018	23353/205545	10.00	NA	49.8	Survey questionnaire of the NHIS	Survey questionnaire of the NHIS	OR: 1.36 (1.27–1.46)	8
Hsu et al. 2019	Cross-sectional	USA	2002–2012	91701/68398663	1.47	28.6 ± 0.21	46.7	ICD-9-CM code 691.8	ICD-9-CM and DRG codes	OR: 0.90 (0.79–1.04)	7
Huang et al. 2021	Cross-sectional	USA	2017.1–2017.12	86969/116564	NA	5.3 ± 5.1	47.3	ICD 10 code L20	ICD 10 codes	OR: 1.11 (1.06–1.16)	7
Huang et al. 2024	Cross-sectional	USA	2005–2018	1376/9884	13.66	9.13 ± 5.19	50.30	Survey questionnaire of the NHIS	Survey questionnaire of the NHIS	OR: 1.45 (1.06–1.99)	7
Jackson-Cowan et al. 2021	Cross-sectional	USA	2008–2018	13398/96084	10.78	NA	50.7	Survey questionnaire of the NHIS	Survey questionnaire of the NHIS	OR: 1.31 (1.20–1.42)	7
Johansson et al. 2017	Cohort	Sweden	1994.2–1996.12 (follow up16 years)	1178/2428	4.90	NA	NA	Parental questionnaire	Medication record of the Swedish Drug Register	OR: 1.12 (0.80–1.56)	9
Kim et al. 2023	Cohort	Korea	2008–2012	30557/89452	NA	NA	49.1	ICD 10 code L20.9	ICD 10 codes	OR: 1.29 (1.07–1.60)	7
Kuniyoshi et al. 2018	Cross-sectional	Japan	2014–2015	1641/8313	NA	NA	NA	ISAAC questionnaire	SDQ	OR: 1.23 (1.04–1.45)	7
Lee et al. 2016	Cohort	China	1998.1–2008.12	18473/18473	4.94**^†^**	NA	53.8	ICD-9-CM code 691 or 691.8	ICD-9-CM code 314	HR: 2.92 (2.48–3.45)	9
Liao et al. 2016	Cohort	China	2000–2010.12	387262/387262	3.70**^††^**	0.71 ± 0.53	46.5	ICD-9-CM 691	ICD-9-CM code 314	HR: 1.16 (1.13–1.19)	9
Lin et al. 2016	Cross-sectional	China	2010	251/2645	NA	NA	NA	ISAAC questionnaire	SNAP-26 questionnaire	OR: 1.66 (1.23–2.25)	8
Radtke et al. 2017	Cross-sectional	Germany	2009	48140/1301531	0.54	NA	NA	ICD 10 code L20	ICD 10 codes	OR: 1.97 (1.74–2.24)	6
Riis et al. 2016	Cohort	Danish	1995.1–2010.1	11877/118751	NA	NA	57.9	Hospital-based medical records of DNRP	DPCR medical records	HR: 1.3 (1.2–1.5)	9
Roh et al. 2022	Cross-sectional	USA	2017.1–2017.12	39779/353743	NA	42.5 ± 13.7	63.9	ICD 10 code L20	ICD 10 codes	OR: 1.05 (0.99–1.10)	7
(a) ADHD as the outcome with AD as the exposure											
Study	Study type	Country	Study period	No. of participants (AD/non-AD)	Prevalence/Incidence (%)	AD patients’ characteristics		Method assessment		Reported effect	Study quality
						Age, years	Gender (female %)	Method assessment AD	Method assessment ADHD		
Romanos et al. 2010	Cross-sectional	Germany	2003–2006	1,952/11,366	NA	9.9 ± 4.1	50.9	Medical examination	Medical or psychological examination	OR: 1.54 (1.24–1.93)	8
Schmitt et al. 2009	Case-control	Germany	2003–2004	1,436/1,436	5.15	12.6 ± 3.8	59.9	ICD 10 code L20	ICD 10 code F90	OR: 1.47 (1.01–2.15)	7
Schmitt et al. 2010	Cohort	Germany	1995–1998 (follow up 10 years)	780/2,136	10.00	NA	47.7	Parental questionnaire of physician diagnosis	SDQ	OR: 1.25 (0.97–1.61)	9
Schmitt et al. 2011	Cohort	Germany	1997–1999 (follow up 10 years)	385/1,193	10.90	NA	49.9	Parental questionnaire of physician diagnosis	SDQ	OR: 2.12 (1.34–3.37)	9
Shrestha et al. 2017	Cohort	USA	2010.1–2015.9	119,716/119,716	NA	51.02 ± 12.51	61.7	ICD-9-CM code 691.8	ICD 9 codes	OR: 1.39 (1.26–1.51)^a^	9
Shyu et al. 2012	Cohort	China	2005.1–2005.12	10,620/178,093	NA	NA	NA	ICD 9 code 691	ICD 9 code 314	OR: 0.73 (0.48–1.09)	7
Strom et al. 2016	Cross-sectional	USA	1997–2013	38,348/348,528	9.28	NA	NA	Survey questionnaire of the NSCH and NHIS	Survey questionnaire of the NSCH and NHIS	OR: 1.14 (1.03–1.26)	7
Vittrup et al. 2021	Cohort	Denmark	1995.1–2012.12 (follow up until 2017.12)	14,283/142,830	7.64 (6.40–9.11)**^†^**	1.92 ± 2.31	43.0	DNPR diagnostic codes (ICD 10 code L20)	DNPR diagnostic codes (ICD 10 code F90)	HR: 1.65 (1.33–2.05)	9
Wan et al. 2020	Cross-sectional	USA	2013.1–2017.12	6,807,687/50,919,169	14.9	10.08 (9.9–10.2)*	51.4	Caregiver report	SDQ	OR: 1.34 (1.24–1.43)	7
Wan et al. 2023	Cohort	UK	1994–2015.2	409,431/1,809,029	1.07 (0.95–1.20)**^†^**	5.00 ± 5.19	48.2	THIN diagnostic codes	Diagnosis codes	HR: 1.02 (0.97–1.06)	9
Wan et al. 2024	Cohort	UK	1994–2015.2	625,083/2,678,888	0.05 (0.04–0.06)**^†^**	47.67 ± 25.20	60.19	Diagnosis and therapy codes	Diagnosis codes	HR: 1.23 (1.04–1.45)	9
Wong et al. 2022	Cross-sectional	China	NA	86/510	NA	NA	NA	Parental questionnaire of physician diagnosis	Parental questionnaire of physician diagnosis	OR: 0.60 (0.20–1.81)	5
Yaghmaie et al. 2013	Cross-sectional	USA	2007	10,408/69,095	9.57	NA	48.2	Parental report of physician diagnosis	Parental report of physician diagnosis	OR: 1.87 (1.54–2.27)	6
Yang et al. 2018	Cross-sectional	China	2010	411/2,361	NA	NA	NA	ISAAC questionnaire	DSM-IV criteria	OR: 1.71 (0.44–06.60)	7
Zhang et al. 2023	Cross-sectional	Netherlands	2006–2013	5,196/51,174	1.20	52.5 ± 11.9	71.7	Self-reported physician-diagnosed AD	Self-report with the M.I.N.I.	OR: 1.46 (1.06–2.00)	9
(b) AD as the outcome with ADHD as the exposure											
Study	Study type	Country	Study period	No. of participants (ADHD/ non-ADHD)	Prevalence	ADHD patients’ characteristics		Method assessment		Reported Effect	Study quality
						Age, years	Gender (female %)	Method assessment AD	Method assessment ADHD		
Akmatov et al. 2021	Case-control	Germany	2017	258,662/2,327,958	7.6	NA	24.4	ICD 10 code L20	ICD 10 code F90	OR: 2.19 (2.16–2.23)	8
Chang et al. 2019	Cohort	China	1980–2010	15,122/100,850	8.8	14.5 ± 4.7	19.3	ICD-9-CM code 691	ICD-9-CM code 314	RR: 1.04 (0.98–1.10)	9
Chen et al. 2017	Case-control	China	1996.1–2010.12	8,201/32,804	17.9	15.68 ± 7.52	24	ICD-9-CM code 691 or 691.8	ICD-9-CM code 314	OR: 1.53 (1.42–1.64)	8
Hak et al. 2013	Case-control	UK	1996–2006	884/3,536	8.1	9.6 ± 2.6	0	Disease code M111.00	Disease code E2E0100	OR: 1.3 (0.9–1.7)	8
Li et al. 2022	Cohort	China	2004–2016	1,386,260 (total)	20	9.7 ± 2.3	52.2	ICD-9-CM code 691; ICD 10 code L20	ICD-9 code 299 or 314; ICD-10 code F84 or F90; more than two outpatient diagnoses given by psychiatrists	HR: 1.34 (1.31–1.37)	9
Osman et al. 2024	Cohort	USA	2003.1–2023.1	NA	Male: 3.06; female: 3.37	NA	NA	ICD 10 code L20	ICD 10 code F90	OR: male: 1.28 (1.24–1.32) female:1.29 (1.24–1.34)	9
Qu et al. 2022	Cohort	USA	2003–2015	423/1,273	39.2	NA	28.1	ICD-9 codes 691 or ICD-10 codes L20	ICD-9 codes 314 or ICD-10 codes F90	OR: 1.71 (1.33–2.19)	9
Suwan et al. 2011	Case-control	Thailand	2010.1–2010.11	40/40	12.5	9.1 ± 2.1	22.5	History and physical examination	DSM-IV criteria	OR: 1.18 (0.27–5.03)	8
Takaesu et al. 2024	Cross-sectional	Japan	2013.6–2021.9	15,028/74,796	8.9	33.1 ± 11.4	39.3	ICD 10 code L20	ICD 10 code F90	OR: 1.54 (1.45–1.64)	6
Tsai et al. 2013	Case-control	China	2002–2009	4,692/18,768	7.2	8.91 ± 3.02	22.1	ICD-9-CM code 691.8	ICD-9-CM code 314	OR: 1.40 (1.22–1.61)	8
Van Der Schans et al. 2016	Case-control	Netherlands	1985.1–2007.12	4,257/17,028	4	8.3	23.3	ATC code D07; ICPC1 code S87	ATC code N06BA04	OR: 1.3 (1.1–1.5)	8
Wang et al. 2018	Case-control	China	NA	216/216	20.4	9.2 ± 1.7	14	ISAAC questionnaire	DSM-IV-TR	OR: 1.72 (1.02–2.88)	8
Zaitsu et al. 2022	Cross-sectional	Japan	2018.4–2019.3	67/691	26.9	Lower grades: 6.9 (6.0–8.0); Upper grades: 11.0 (10.0–12.0)	Lower grades: 44.8;Upper grades: 39.4	ICD 10 code L20	ICD 10 code F90	OR: Lower grades: 0.87 (0.23-3.33);Upper grades: 5.06 (1.28-20.05)	7

Abbreviations: AD = atopic dermatitis; ADHD = attention deficit hyperactivity disorder; No.= number; CI = confidence interval; SD = standard deviation; NA = not applicable; HR = hazard ratio; RR = risk ratio; OR = odd ratio; ICD = International Classification of Diseases; ICD-9-CM = International Classification of Disease 9th edition Clinical Modification; ISAAC = International Study of Asthma and Allergies in Childhood; SDQ = Strengths and Difficulties Questionnaire; DRG = Diagnosis-Related Groups; ECI-4 = Early Childhood Inventory-4; NHIS = National Health Interview Survey; NSCH = National Survey of Children’s Health; DNPR = Danish National Patient Registry; DPCR = Danish Psychiatric Central Registry; THIN = The Health Improvement Network; DSM = Diagnostic and Statistical Manual of Mental Disorders; M.I.N.I.= Mini International Neuropsychiatric Interview; ICPC = the International Classification for Primary Care; ATC = the Anatomical Therapeutic Chemical classification.

^†^ Incidence rates of outcomes (per 1000 person-years).

^††^ Cumulative incidence.

* Mean (95% CI).

^a^ Shrestha et al. reported OR in Commercial: 1.39 (1.26–1.51); Medicare: 1.75 (1.12–2.65); Medi-Cal: 1.74 (1.13–2.75), respectively.

### 3.2. Study quality

Studies included were classified as moderate to high quality. All but one of the studies scored above 7 on the NOS [35]. Cross-sectional studies scored ranges from 5 to 9 using the AQHR tool (Table S1).

### 3.3. Risk of ADHD in patients with AD

Among the 30 studies reporting OR, we found patients with AD had an overall statistically significant increased risk of ADHD (ORs = 1.34, 95% CI 1.25–1.44, *I*^2^ =86.8%, *p* < 0.01; Figure 2a) [4,14–17, 24–26,35–56] compared to those without AD. The pooled estimates in six studies reporting HR also indicated a moderately positive association (HRs =1.42, 95% CI 1.20-1.68, *I*^2^ =97.0%, *p* < 0.01; Figure 2b) [57–62]. Among total 7,118,647 patients with AD, 1,027,334 patients were diagnosed with ADHD. The pooled prevalence in the 19 studies reporting prevalence was 7.9% (95% CI 3.8–12.0, Figure S1). Despite significant heterogeneity, the results remained consistent across employing the leave-one-out method, confirming their robustness (Figure S2). Notably, excluding Lee et al. [57] from the analysis led to a reduction in the combined HR to 1.21, suggesting potential recall bias linked to the study’s retrospective design and the young age of the cohort. Visual assessment of the funnel plot revealed no evidence of publication bias (Figure S3), corroborated by Egger’s test (*t* = 1.61, *p* = 0.118).

Figure 2.(a). Forest plot of ORs for the AD and the risk of ADHD. Shrestha et al. reported or in commercial: 1.39 (1.26–1.51); Medicare: 1.75 (1.12–2.65); Medi-Cal: 1.74 (1.13–2.75). ORs from Strom et al. and Huang et al. are reported by age separately. (b). Forest plot of HRs for the AD and the risk of ADHD.

**Figure:**
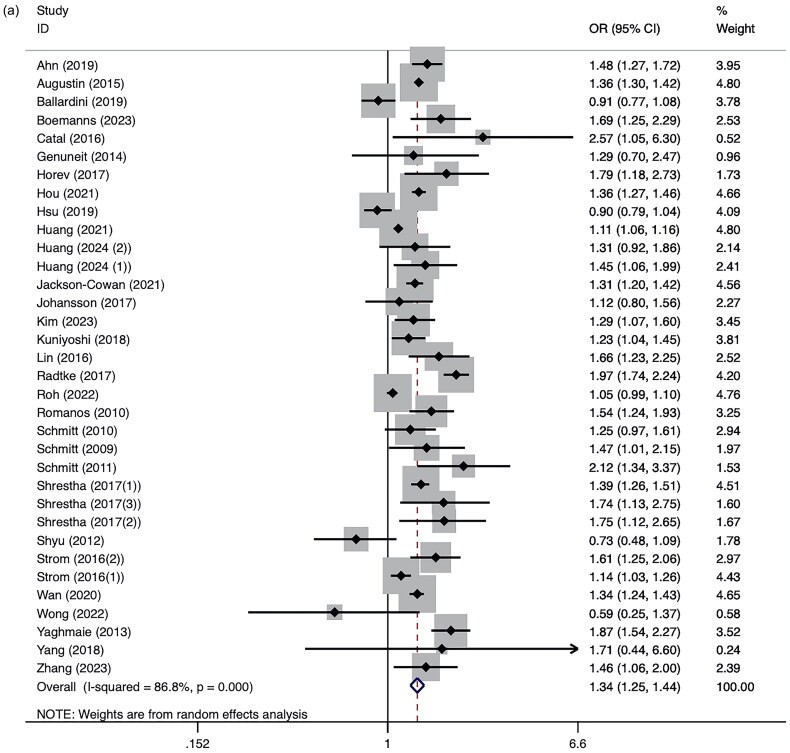

**Figure:**
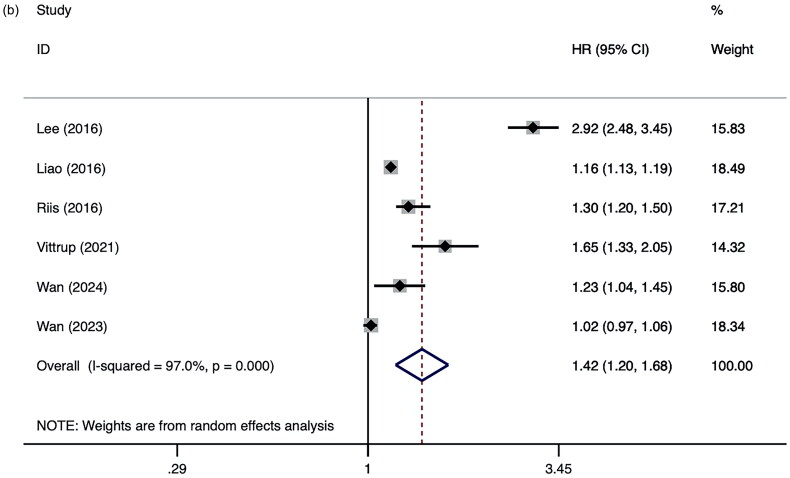

### 3.4. Risk of AD in patients with ADHD

**Figure 3.**
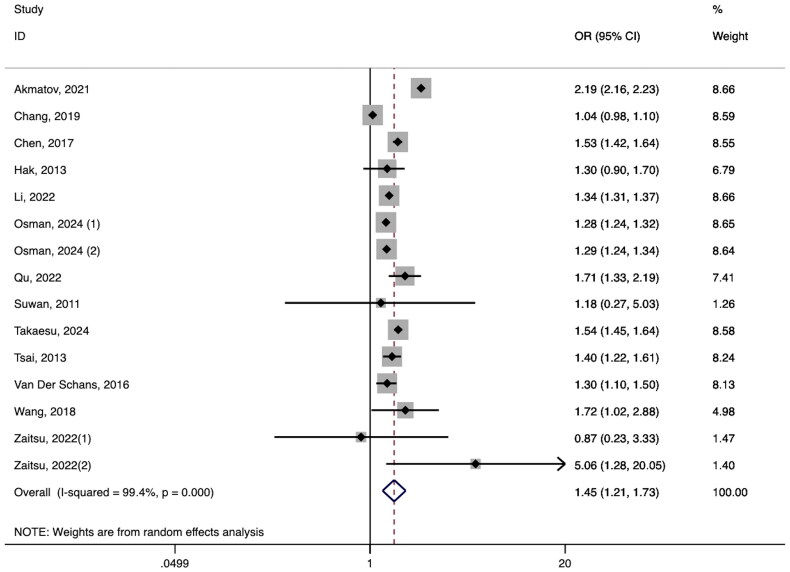
ADHD and the risk of AD. Osman et al. reported or in males and females respectively. Zaitsu et al. reported or in lower grades and upper grades respectively.

The synthesis of findings from thirteen studies indicated a heightened prevalence of AD in patients with ADHD (ORs = 1.45, 95% CI 1.21–1.73, *I*^2^ = 99.4%, *p* < 0.01, Figure 3) [18–21,63–72]. Among a total of 2,233,275 patients with ADHD, 318,609 patients were diagnosed with AD. The pooled prevalence in the above studies reporting the prevalence of AD in patients with ADHD was 11.9% (95% CI 7.4–16.3; Figure S4). Despite significant heterogeneity, excluding any single study did not significantly impact the overall conclusions (Figure S5). Funnel plot and Egger’s test (*t* = −1.13, *p* = 0.279) revealed no evidence of publication bias (Figure S6).

### 3.5. Subgroup analysis

To further explore the reciprocal relationship between AD and ADHD, we carried out subgroups (Table 2) and meta-regression (Table S2).

**Table 2.** Bidirectional association between AD and ADHD according to subgroups.

Subgroups	*N*	Effect size OR (95% CI)	Test (s) of heterogeneity	
			*I* ^2^	*P*-value
**Risk of ADHD in patients with AD**	30	1.34 (1.25–1.44)	86.8%	<0.1
Region				
Asia	10	1.25 (1.03–1.51)	75.6%	<0.1
Europe	10	1.51 (1.31–1.73)	75.9%	<0.1
North America	10	1.29 (1.19–1.41)	89.4%	<0.1
Study type				
Cross-sectional	19	1.35 (1.25–1.46)	90.4%	<0.1
Cohort	8	1.27 (1.07–1.49)	74.4%	<0.1
Case-control	3	1.67 (1.28–2.19)	0.0%	0.489
Sample size				
≤1000	3	1.24 (0.59–2.63)	63.5%	<0.1
1000–10,000	10	1.41 (1.27–1.57)	18.7%	0.265
>10,000	17	1.31 (1.21–1.42)	91.7%	<0.1
Study period, years				
≤1	12	1.39 (1.21–1.59)	93.3%	<0.1
>1, ≤10	8	1.31 (1.13–1.50)	83.5%	<0.1
>10	8	1.33 (1.24–1.42)	41.4%	<0.1
Assessment of AD				
Diagnosed by a doctor/professional	13	1.34 (1.20–1.49)	92.3%	<0.1
Standardized questionnaires	4	1.22 (0.91–1.63)	78.4%	<0.1
Self/parental/caregiver’s report of doctoral diagnosis	13	1.38 (1.27–1.48)	60.4%	<0.1
Assessment of ADHD				
Diagnosed by a doctor/professional	14	1.32 (1.18–1.47)	91.7%	<0.1
Standardized questionnaires	8	1.29 (1.09–1.54)	77.0%	<0.1
Self/parental/caregiver’s report of doctoral diagnosis	8	1.41 (1.28–1.55)	66.2%	<0.1
Age				
<18	25	1.30 (1.22–1.40)	78.7%	<0.1
≥18	6	1.63 (1.29–2.07)	95.0%	<0.1
Gender				
Predominantly female (> 50%)	8	1.40 (1.25–1.57)	85.1%	<0.1
Predominantly male (<50%)	9	1.21 (1.05–1.39)	87.0%	<0.1
AD severity				
Mild-moderate AD	6	1.33 (1.10–1.60)	70.7%	<0.1
Severe	5	2.62 (1.76–3.92)	77.7%	<0.1
Atopic comorbidity^†^	3	1.42 (1.11–1.81)	89.7%	<0.1
Number of allergic disorders (*n* = 1)	2	1.71 (1.10–2.66)	71.4%	<0.1
Number of allergic disorders (*n* ≥ 2)	2	2.89 (1.18–7.10)	84.1%	<0.1
Sleep disorder, yes versus no	3	2.43 (2.14–2.76)	0.0%	0.803
**Risk of AD in patients with ADHD**	13	1.45 (1.21–1.73)	99.4%	<0.1
Region				
Asia	8	1.38 (1.21–1.56)	92.8%	<0.1
Europe	3	1.57 (1.03–2.40)	96.2%	<0.1
North America	2	1.30 (1.24–1.36)	61.9%	<0.1
Study type				
Cross-sectional	2	1.71 (0.87–3.38)	44.0%	0.168
Cohort	4	1.26 (1.71–1.36)	94.3%	<0.1
Case-control	7	1.54 (1.21–1.96)	96.6%	<0.1
Sample size				
≤1000	3	1.73 (1.00–2.99)	17.1%	0.306
1000–10,000	2	1.52 (1.16–1.98)	43.4%	0.184
>10,000	7	1.45 (1.12–1.86)	99.6%	<0.1
Study period, years				
≤1	3	2.05 (1.29–3.26)	23.9%	0.268
>1, ≤10	3	1.49 (1.39–1.61)	16.2%	0.303
>10	6	1.31 (1.22–1.40)	93.4%	<0.1

Abbreviations: AD = atopic dermatitis; ADHD = attention deficit hyperactivity disorder; N = number; CI = confidence interval; OR = odd ratio.

**^†^**AD plus asthma/allergic conjunctivitis/allergic rhinitis/hay fever/food allergy.

#### 3.5.1. Geographical region

In studies investigating the risk of ADHD in patients with AD, the link seems stronger in Europe (ORs = 1.51, 95% CI 1.31–1.73) than in Asia (ORs = 1.25, 95% CI 1.03–1.51) and North America (ORs = 1.29, 95% CI 1.19–1.41). When examining ADHD as an exposure factor and AD as an outcome measure, the link also stronger in Europe (ORs = 1.57, 95% CI 1.03–2.40) than in Asia (ORs = 1.38, 95% CI 1.21–1.56) and North America (ORs = 1.30, 95% CI 1.24–1.36). Our findings aligned with prior research [50]. Racial demographics and healthcare quality in these regions might contribute to this variability [42].

#### 3.5.2. Study type

A subgroup analysis by study type demonstrated consistent findings across cross-sectional (ORs= 1.35, 95% CI 1.25–1.46) and cohort studies (ORs= 1.27, 95% CI 1.07–1.49). Specifically, case-control studies indicated an increased risk of ADHD among patients with AD (ORs= 1.67, 95% CI 1.28–2.19). Furthermore, for studies examining the risk of AD in patients with ADHD, the ORs for the case-control, cohort studies and cross-sectional group were 1.54 (95% CI 1.21–1.96), 1.26 (95% CI 1.71–1.36), 1.71 (95% CI 0.87–3.38), respectively. The higher ORs observed in case-control studies may be attributed to sample size, as smaller samples were prone to producing exaggerated estimates. Additionally, the elevated OR in the cross-sectional group could be influenced by the findings from Zaitsu et al. [72], where the ADHD sample size was fewer than 50 individuals.

#### 3.5.3. Sample size

Subgroup analysis based on sample size revealed consistent results across groups with a sample size of 1000–10000 (ORs = 1.41, 95% CI 1.27–1.57) and >10000 (ORs = 1.31, 95% CI 1.21–1.42), while no significant association was shown in group with sample ≤1000 (ORs = 1.24, 95% CI 0.59–2.63). For studies investigating the risk of AD in ADHD patients, the results were as follows: ≤1000 (ORs = 1.73, 95% CI 1.00–2.99), 1000-10000 (ORs = 1.52, 95% CI 1.16–1.98) and > 10000 (ORs = 1.45, 95% CI 1.12–1.86).

#### 3.5.4. Study period

Our analysis stratified the study period into three groups: ≤1 year, 1–10 years, and >10 years. In studies where AD was considered as the exposure, we revealed consistent results across groups with the study period ≤1 year (ORs = 1.39, 95% CI 1.21–1.59), 1–10 years (ORs= 1.31, 95% CI 1.13–1.50), and >10 years (ORs = 1.33, 95% CI 1.24–1.42), respectively. Analogously, in studies where ADHD was considered as the exposure, the result revealed that studies with shorter periods were more likely to report higher OR values (ORs = 2.05, 95% CI 1.29–3.26). The ORs for the study period of 1–10 years and >10 years were 1.49 (95% CI 1.39–1.61) and 1.31 (95% CI 1.22–1.40). The study period also influenced the pooled effect, as further validated by meta-regression (*p* = 0.21). This may be because longer-duration studies could dilute the observed effect as a result of the influence of additional factors, such as therapeutic interventions, comorbidities, or environmental changes over time.

#### 3.5.5. Assessment of AD and ADHD

The assessment through physician-diagnosed but self/parent/caregiver-reported appears to be associated with a higher frequency of ADHD (ORs = 1.38, 95% CI 1.27–1.48; ORs = 1.41, 95% CI 1.28–1.55) compared to other diagnostic methods. Among thirteen studies investigating the risk of AD in patients with ADHD, twelve studies utilized the ICD codes while one employed the standardized questionnaire.

#### 3.5.6. Age

Patients diagnosed with AD were stratified into two age groups: <18 years and ≥ 18 years. The odds of ADHD were higher in studies enrolling adults (ORs = 1.63, 95% CI 1.29–2.07) than those evaluating children and adolescents (ORs = 1.30, 95% CI 1.22–1.40). In studies investigating the risk of AD in ADHD patients, all but one article [69] examined individuals younger than 18 years old.

#### 3.5.7. Gender

Predominantly female studies (female ratio > 50%) reported that patients with AD were more likely to develop ADHD (ORs= 1.40, 95% CI 1.25–1.57) than those dominated by males (ORs = 1.21, 95% CI 1.05–1.39). While patients with AD were considerably higher in females [2], ADHD was historically known as male dominant disorder [73]. Thus, it is unsurprising that the majority of studies considering ADHD as an exposure included predominantly male participants. Nonetheless, Chang et al. [64] reported an increased risk of AD in female siblings with ADHD after stratification by sex which may due to different impacts of the immune system on neurodevelopment influenced by sex hormones [42]. Notably, recent research proved that ADHD also impaired females equally, especially in social functioning, time perception, stress tackling and mood disorder [74].

#### 3.5.8. AD severity

Data from six eligible studies were incorporated into this analysis. The findings revealed that patients with severe AD (ORs= 2.62; 95% CI: 1.76–3.92) had a higher propensity to develop ADHD than mild-to-moderate AD (ORs = 1.32, 95% CI: 1.10–1.60). Meta-regression further confirmed that the AD severity significantly impacted the combined effect (*p* = 0.006).

#### 3.5.9. Comorbidity

Three studies provided data on sleep disturbance reported that AD with related sleep disturbance tends to be ADHD (ORs = 2.43, 95% CI 2.14–2.76). Furthermore, AD accompanied by other allergic diseases was associated with increased odds of ADHD (ORs = 1.42, 95% CI 1.11–1.81). Besides, having two or more additional allergic diseases was rather higher (ORs = 2.89, 95% CI 1.18–7.10) than only having one additional allergic disease (ORs = 1.71, 95% CI 1.10–2.66).

## 4. Discussion

This systematic review rigorously examines existing evidence concerning the bidirectional association between AD and ADHD within observational studies. Our analysis revealed that individuals with AD have a 1.34-fold increased risk of developing ADHD. Moreover, our analysis also found that the prevalence of AD and the risk of its development were elevated in patients with ADHD, suggesting a potential bidirectional association between AD and ADHD.

Previous research, such as the review by Schmitt et al. [75], has recognized AD as an independent risk factor for ADHD. However, most studies relied on parent reports or secondary data, which lack reliability. Schans et al. [22] also established AD as a potential risk factor for ADHD, yet the limited number of included studies and insufficient evidence weakened the conclusions, particularly did not analyze AD severity and the bidirectional relationship between AD and ADHD. Although the meta-analysis conducted by Cheng et al. [76] included more studies and analyze AD severity, it failed to explore the bidirectional relationship between these two disorders. Our study synthesizes prior research findings and incorporates novel studies to offer deeper insights into the bidirectional association between these two disorders. Furthermore, we discovered that severe AD, along with sleep disorder and other atopic comorbidity, significantly increased susceptibility to ADHD. Below, we propose several hypotheses that could plausibly account for our observations.

Predominantly, immune dysregulation and inflammatory disorders are the key players between AD and ADHD [77]. In the pathogenesis of AD, inflammatory cytokines provoked by allergic reactions may disrupt the development and maturation of specific brain regions like the prefrontal cortex (PFC) and anterior cingulate cortex (ACC) [78], potentially leading to cognitive dysfunctions that precipitate ADHD [79–81]. Beyond the direct impact on the PFC and ACC, cytokines like IL-6, TNF-α might also indirectly modulate neuronal activity by activating the hypothalamus-pituitary-adrenal (HPA) axis [82], elevating glucocorticoid (GC) levels that influence these regions [83]. Furthermore, inflammatory factors may compromise the integrity of the blood-brain barrier, facilitating the infiltration of substances that could trigger alterations in neurotransmitter metabolism associated with ADHD [84]. Notably, we found that severe AD was closely associated with ADHD, consistent with findings from Cheng et al. [76] This may be attributed to heightened inflammation, as aforementioned neuro-immune mechanisms explained. Additionally, persistent chronic skin symptoms may lead to more severe sleep disturbances and psychological stress, while the use of multiple systemic medications could further exacerbate this association [79,85]. Sleep disturbance, another significant contributing factor, could amplify the likelihood of both AD and ADHD [11,86]. Insufficient sleep, often a consequence of AD [87], can impair brain development through the aforementioned neuro-immunomodulatory mechanisms, thereby exacerbating the symptoms of inattention and hyperactivity [88,89] and perpetuating a detrimental cycle of both conditions [50]. Furthermore, patients with multiple atopic comorbidities face a heightened risk of subsequently developing ADHD, as illustrated by Lee et al. [57] Allergic comorbidities such as asthma, allergic arthritis and hay fever are separately associated with increased ADHD [22,39,90]. This suggests a cumulative effect of allergic comorbidities on mental health disorders.

Recent studies have discovered the genetic associations between AD and ADHD [14,68,77]. Children with a family history of allergies are at a higher risk of developing ADHD [20,21], suggesting that both disorders may share genetic markers, particularly those related to immune regulation and neural development [82]. Epigenetic mechanisms might elucidate how the environment influences gene expression in these diseases [91,92], highlighted by observed hypomethylation in patients [93,94].

Our study possesses several advantages. Firstly, it provides an extensive analysis of the bidirectional relationship between AD and ADHD, and also explores the potential mediated factors (severe AD, sleep problems, and multiple allergic diseases). Second, most of the studies we included were based on population studies with large sample sizes. All included association data were multivariate adjusted effect estimates (OR, RR, HR and 95% CI), which effectively controlled for confounding factors such as age and gender. For outcome measures, we analyzed ORs and HRs independently to assess the consistency across different types of studies.

However, several limitations were also noted. First, discrepancies in diagnostic criteria across studies should be mentioned. Although Hanifin and Rajka criteria [1] and the Diagnostic and Statistical Manual of Mental Disorders (DSM) criteria [5] have been recommended for AD and ADHD diagnoses respectively, their application in population-based studies poses challenges. Most studies included have relied on diagnostic codes and questionnaires. Currently, International Classification of Diseases (ICD) codes have been validated for accurate diagnosis of AD and ADHD [5]. Our analysis revealed that the AD-ADHD relationship was weaker when research was restricted to the use of diagnostic codes (ORs = 1.29). This may stem from the methodological constraints associated with employing the ICD codes, including issues such as over-generalization and inconsistencies across different healthcare delivery settings [15,95]. Furthermore, standard questionnaires like the International Study of Asthma and Allergies in Childhood (ISAAC) questionnaire [96] and the Strengths and Difficulties Questionnaire (SDQ) [97] have been proven effective and widely used in research but often depend on parental reports, introducing recall bias and potential for exaggerated reports due to parental stress. Utilizing these questionnaires alone can easily result in both false positives and negatives [98], highlighting the challenges in ADHD diagnosis, which is considered reliable when confirmed by clinicians [1]. Moreover, the potential for physician misdiagnosis must not be overlooked. Given these diagnostic complexities, identifying a consistent and reliable diagnostic method is crucial. Second, the limited number of relevant studies included in our analysis restricted our ability to perform a comprehensive pooled analysis of some risk factors, including sociodemographic (e.g. family history, economic education and maternal smoking history) and clinical factors (e.g. age at AD onset and duration of AD, medications received, serum levels and non-allergic comorbidities). Therefore, there is a pressing need for further research into these critical variables to investigate the underlying mechanisms of the mutual associations between AD and ADHD, as well as other psychiatric disorders. Third, while well-established in children, the link between AD and ADHD in adults lacks sufficient evidence [23]. Both AD and ADHD typically manifest during infancy and early childhood, and are intricately linked to early neurodevelopmental processes as demonstrated in a recent epidemiological study [2,82]. Consequently, most research has focused on pediatric populations, with adult ADHD and AD being comparatively underexplored in epidemiological studies. Longitudinal studies indicate that both diseases often evolve and persist into adulthood, with substantial prevalence rates among adults [75,99–101]. Moreover, comorbid psychiatric conditions are more prevalent in adult ADHD populations [102]. Intriguingly, we found that the risk of ADHD in AD adults was higher compared to younger age groups, underscoring the importance of updating global epidemiological estimates for adult ADHD and AD. Thus, there is a pressing need for further research in adult cohorts to improve diagnosis and management. Additionally, significant *I*^2^ typically generated by large-scale research underscores the inherent limitation of meta-analyses based on observational studies [103,104].

## 5. Conclusion

This meta-analysis indicates the reciprocal relationship between AD and ADHD. The study emphasizes the importance of interdisciplinary consultations to integrate these findings into clinical practice, thus improving mental health care and preventing health deterioration. Furthermore, to enhance physicians’ ability to identify illnesses early and develop effective preventive plans, standardization of diagnostic criteria and additional research into mediating variables are needed, especially in individuals with severe AD manifestations, sleep problems, and multiple allergic diseases.

## Data Availability

All data were extracted from previously published studies or openly available data sets that are therefore publicly available. And the data supporting the present study’s findings can be accessed in this manuscript and its supplementary files. Detailed data are available from the corresponding author on reasonable request.
